# Pyrrole and Fused Pyrrole Compounds with Bioactivity against Inflammatory Mediators

**DOI:** 10.3390/molecules22030461

**Published:** 2017-03-17

**Authors:** Samar Said Fatahala, Sherifa Hasabelnaby, Ayman Goudah, Ghada I. Mahmoud, Rania Helmy Abd-El Hameed

**Affiliations:** 1 Pharmaceutical Organic Chemistry Department, Faculty of Pharmacy, Helwan University, Ain-Helwan, Helwan 11795, Cairo, Egypt; zeiadomar@yahoo.com; 2 Pharmaceutical Chemistry Department, Faculty of Pharmacy, Helwan University, Ain-Helwan, Helwan 11795, Cairo, Egypt; sherifajanaa@yahoo.com; 3 Department of Chemistry and Biochemistry, Concordia University, 7141 rue Sherbrooke O., Montréal, H4Bm1R6 QC, Canada; 4 Department of Pharmacology, Faculty of Veterinary Medicine, Cairo University, Giza/Egypt, Giza 12211, Egypt; aymangouda@yahoo.com; 5 Department of Biochemistry, Faculty of Agriculture, Cairo University, Giza/Egypt, Giza 12211, Egypt; ssfathallah@yahoo.com

**Keywords:** pyrroles, pyrrolopyridines, synthesis, anti-inflammatory assay, cytokine inhibitors, docking study

## Abstract

A new series of pyrrolopyridines and pyrrolopyridopyrimidines have been synthesized from aminocyanopyrroles. The synthesized compounds have been characterized by FTIR, ^1^H-NMR and mass spectroscopy. The final compounds have been screened for in vitro pro-inflammatory cytokine inhibitory and in vivo anti-inflammatory activity. The biological results revealed that among all tested compounds some fused pyrroles, namely the pyrrolopyridines **3i** and **3l**, show promising activity. A docking study of the active synthesized molecules confirmed the biological results and revealed a new binding pose in the COX-2 binding site.

## 1. Introduction

Non-steroidal anti-inflammatory drugs (NSAIDs) belong to a class of drugs used worldwide for the treatment of pain, fever and inflammatory diseases [1,2,3,4,5,6,7,8]. Recently, use of NSAIDs has been extended [3] to reduce the risk of developing cancer through reduction of inflammation, which is an important risk factor for cancer.

**Figure 1:**
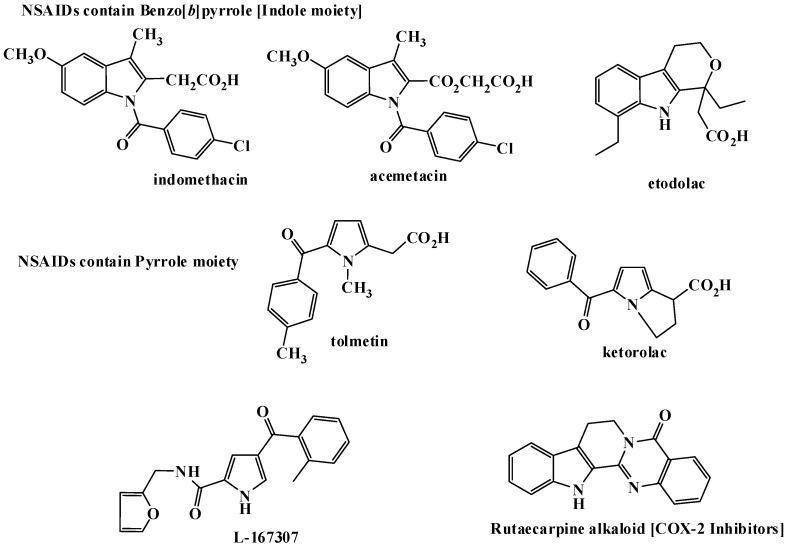
NSAIDS containing pyrroles moiety.

Classical NSAIDs such as aspirin, ibuprofen and diclofenac [3,9,10,11] represent some of the most widely prescribed NSAIDs to relieve short-term fever, pain and inflammation. Two isoenzymes of cyclo-oxygenase COX, COX-1 (constitutive form) and COX-2 (inducible form), have been identified [4,12,13]. The classical NSAIDs inhibit both isoenzymes and their use is often accompanied by gastrointestinal intolerance [12], due to a decreased production of protective prostaglandin E2 in the stomach. New drugs that selectively inhibit COX-2 exhibit a better gastric tolerance profile [5,9,11,14,15,16,17,18]. Among well-known NSAIDs, pyrrole ring derivatives [19] are of remarkable interest [20,21,22,23]. Examples are benzo[*b*]pyrrole derivatives such as indomethacin (Indacin^®^), acemetacin (Emflex^®^), and etodolac (Etodine^®^) and pyrrole derivatives like tolmetin (Rumatol^®^) and ketorolac (Ketolac^®^) [14,24,25]. These compounds block prostaglandin synthesis by nonselective inhibition of COX-1 and COX-2 (indomethacin, acemetacin, tolmetin, and ketorolac) or by selective inhibition of COX-2 (etodolac). Rutaecarpine, a quinazolinocarboline alkaloid [26,27], has been isolated from some well-known Chinese herbal drugs, namely Wu-Chu-Yu, and Shih-Hu. It exhibits a strong anti-inflammatory activity and has shown potent and selective inhibitory activity against COX-2. A series of *N*-pyrrolylcarboxylic acids have been reported to be potent COX-2 inhibitors [28,29]. Also, a group of Merck researchers have reported [30] that L-167307 (pyrrole derivative) showed potent inhibitory activity towards the pro-inflammatory cytokine TNF-α. Structures of some of these compounds are shown in Figure 1.

Relatively recent discoveries [31] indicate that long-term use of NSAIDs is associated with numerous risk factors, including ulceration and other adverse effects. Moreover, instead of their ability to improve pain and tenderness, NSAIDs did not prevent disease progression in rheumatoid arthritis [5,32,33,34], a disease whose pathogenesis has been linked to the presence of pro-inflammatory cytokines, such as interleukin-1 (IL-1β) or tumor necrosis factor (TNF-α).

Numerous NSAID derivatives [7] have been prepared in order to improve the analgesic/anti-inflammatory activity, minimize side effects, prolong plasma half-life, increase COX-1/COX-2 selectivity, cytostatic activity and change water solubility or lipophilicity [35,36,37]. The search for alternative anti-inflammatory drugs with minimal adverse effects is one of the biggest challenges in modern medicinal chemistry. Here we highlight some aspects of the chemistry of some newly synthesized fused pyrrole derivatives and test them for anti-inflammatory activity. 

## 2. Results

### 2.1. Chemistry

**Scheme 1:**
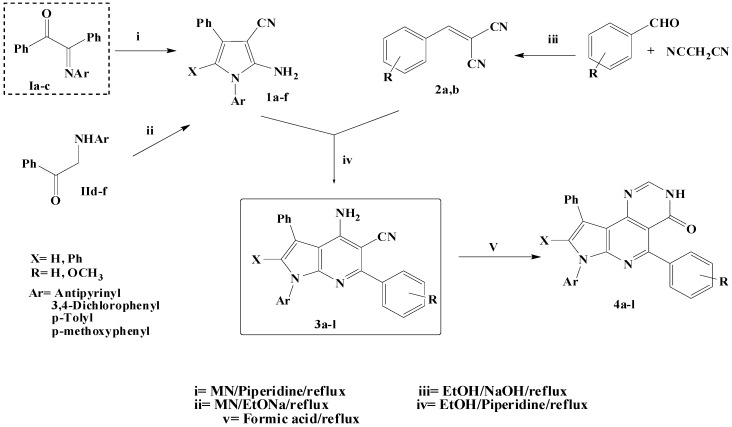
Synthesis of pyrrolopyridines (**3a**–**l**) and fused pyrimidines (**4a**–**l**).

The synthesis of the target compounds is summarized in Scheme 1. To synthesize compounds **3a**–**l** and **4a**–**l**, several 2-aminopyrrole-3-carbonitriles **1a**–**f** were prepared by condensing malononitrile with the two intermediates **I** or **II**, respectively, in strong basic medium [38,39,40,41]. The 2-arylidene malononitriles **2a**,**b** were prepared by condensation of malononitrile with the aromatic aldehydes benzaldhyde or anisaldehyde [42,43,44]. Cyclocondensation of 2-aminopyrrole-3-carbonitriles **1a**–**f** with the 2-arylidene-malononitriles **2a**,**b** in refluxing ethanol containing a catalytic amount of piperidine followed by treating [42] the product with crushed ice/dilute HCl, afforded 4-aminopyrrolopyridine carbonitrile derivatives **3a**–**l** in yields ranging from 39%–94%. The chemical structures of the newly synthesized derivatives **3a**–**l** were established on the basis of analytical and spectral data. Their IR spectra displayed the presence of NH_2_, CN, and C=N absorption bands in the 3316–3467, 2176–2224 and 1568–1618 cm^−1^ regions, respectively, as well as the presence of C=O bands in the 1698–1723 cm^−1^ region for compounds **3c**, **3f**, **3i,** and **3l**. In the ^1^H-NMR spectra, the D_2_O exchangeable signal of the NH_2_ protons was recorded at 4.70–5.21 ppm. The interaction [43,44,45,46] of 4-aminopyrrolopyridine carbonitrile derivatives **3a**–**l** with formic acid led to the formation of corresponding pyrimidin-4-one derivatives **4a**–**l** in yields ranging from 52%–87%. The structures of **4a**–**l** were elucidated on the basis of their spectra (IR, ^1^H-NMR, and MS) and elemental analysis. For example, their IR spectra revealed the absence of NH_2_ and CN absorption bands which are present in the IR spectra of their precursors **3a**–**l**, and also revealed the presence of NH and CO absorption bands in the 3336–3475 and 1682–1729 cm^−1^ regions, respectively. Moreover, the ^1^H-NMR spectra showed the expected aromatic signals as well as two singlet signals near δ 8.20 and 8.45 ppm assignable to the pyrimidine CH=N, and amidic NH protons, respectively. All synthesized compounds are listed in Table 1.

### 2.2. Anti-Inflammatory Assays

Ten of the synthesized compounds (**3b**–**d**, **3g**–**l**, and **4k**) have been evaluated for their anti-inflammatory activity, using a method established by Harrk et al. [47]. Six of the tested compounds induced significant anti-inflammatory activity, comparable with that of diclofenac. Generally, the data listed in Table 2 indicated that the anti-inflammatory activity increased with time and their duration of action persisted for 4 h after the administration of the carrageenan.

Compound **3l** exerted manifestly significant activities compared to a standard drug at all-time intervals post-carrageenan (≈19.97%, ≈30.05%, ≈36.33% and ≈36.61% inhibition at 1st, 2nd, 3rd and 4th hour interval post-carrageenan administration). The activity profile was the same as that of the standard drugs (increasing response with time). Compound **3i** exerted noticeable activities compared to the standard drugs at the 1st, 2nd, 3rd and 4th hour post-carrageenan (≈21.67%, ≈26.04%, ≈29.3% and ≈31.28% inhibition at 1st, 2nd, 3rd and 4th hour interval post-carrageenan). The activity profile was the same as that of the standard drugs (response increasing with time), yet the activity shown by compounds **3b**, **3c**, **3h**, and **3j** over all tested intervals was modest but still significant. Compounds **3c** and **3j** showed increased activity from the second to fourth hours. Compounds **3d**, **3g**, **3k** and **4k** were all inactive over all tested periods and have shown percentages of inhibition from 7% to 14% at the 1st to 4th hour, respectively. Compounds **3b**, **3c**, **3h**, and **3j** exhibited moderate anti-inflammatory activity compared to the standard drugs (response increasing with time) that improved over time, yet the activity shown was small, but still significant, for compounds **3b**, **3c**, **3h** and **3j** over all tested intervals. Compounds **3j** showed increased activity from the second till the fourth hours. On the other hand, the lowest anti-inflammatory activity has been observed in compounds **3d**, **3g**, **3k** and **4k** over all tested periods, showing % inhibitions <7 till 14.48% at the 1st to 4th hour, respectively, and are indicated as inactive.

### 2.3. Docking and Modelling

The free binding energy is directly proportional to the constrained binding constant whereby a higher ΔG value represents good binding. The clash score describes the degree of fitting and if a compound is at an optimum distance from the specified residues or if it is very close. The less the clash score, the more the mode of binding is preferred. The COX-2 enzyme binding site contains a number of residues such as Ser 530, Tyr 355, Trp 387, Arg 120, Glu 524 and Gln 192. The well-known COX-2 inhibitors did not bind to all of these residues at the same time. It has been reported [48] that ibuprofen for example interacts only with Arg 120 with an electrostatic bond. On the other hand diclofenac forms hydrogen bonds with Tyr 355.

In this study a number of substituted pyrrolo[2.3-*b*]pyridine derivatives (**3b**–**d**, **3g**–**l**, and **4k**) have been synthesized and tested for their anti-inflammatory activity. A molecular docking study was done to predict the binding mode of the tested compounds and to interpret their activity.

**Figure 2:**
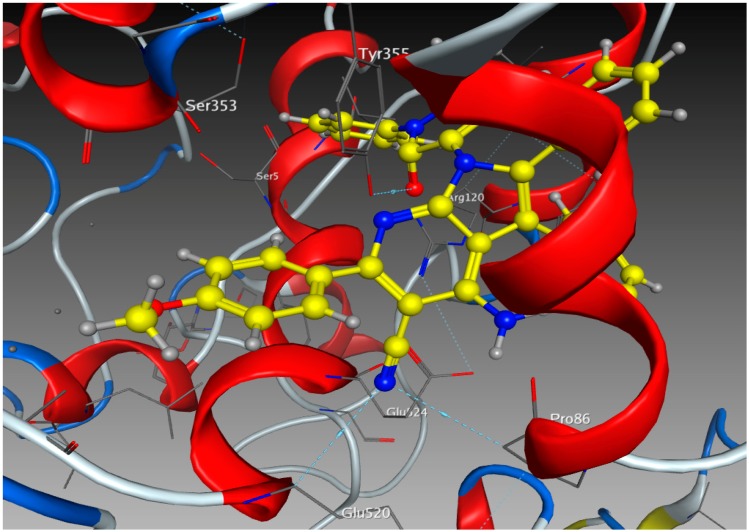
The best binding mode of compound **3l**.

The docking scores almost matched the biological results in the ranking the compounds **3b**, **3c**, **3h**, **3i**, **3j** and **3l**. Compound **3l** (an antipyrinyl derivative) with the top ranked docking score (−12.23) was also the most biologically active (as shown in Table 2 and Table 3). It showed the least clash score (2.51) when compared with the rest of the series and also diclofenac with a 5.2 clash score. The best binding pose of compound **3l** showed a hydrogen bond formed between the C=O of the pyrazole-one moiety and the –OH group of Tyr 355. Furthermore, other interactions by its nitrile group and both Glu 520 and Pro 86 have been observed (Figure 2).

**Figure 3:**
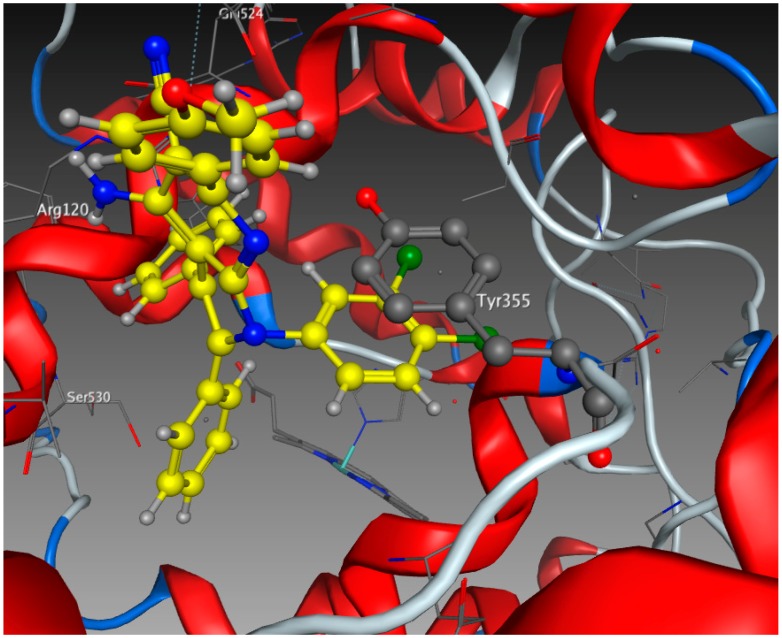
The best binding mode of compound **3j**.

On the other hand compound **3j** (a 3,4-dichlorophenyl derivative) with −11.42 kcal/mol score contains a halo-substituted aromatic ring that encourages the formation of π-π interactions with the aromatic ring of Tyr 355 and that has been illustrated clearly, when the halo-substituted ring was parallel to the aromatic ring of Tyr 355 in one of docked the poses of compound **3j** (Figure 3). From the docking results and observations, the aminopyrrolo[2,3-*b*]pyridine-5-carbonitrile moiety linked with substituted pyrazole-3-one scaffold that was in both compounds **3l** and **3j** would have been a good scaffold with excellent binding to COX-2 and might need further studies for selection of the best substituent groups with high predicted activities. Some of the docked compounds showed a shift from the binding site and interacted with other residues as in the case of compound **4k** (*p*-methoxyphenyl derivative) that showed hydrogen bonds with Ser 119 and Tyr 115 and hence, its clash score was the highest (7.72).

**Figure 4:**
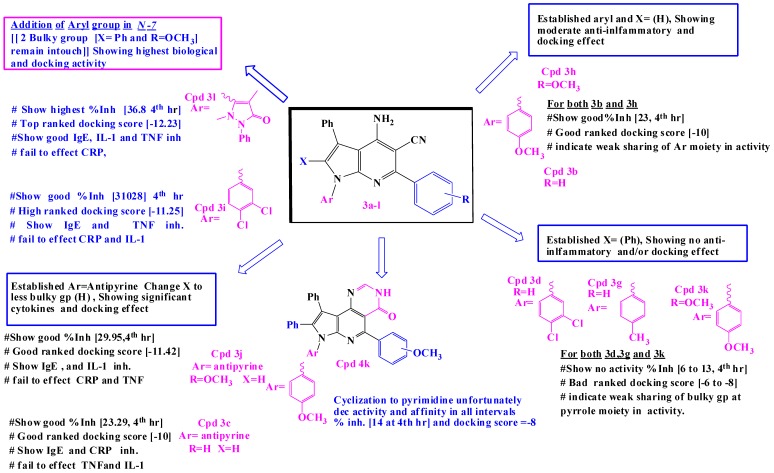
SAR and discussion for all bioassay and docking studies performed on the tested compounds.

The structure activity relationship is illustrated according to all results in Figure 4. The docking results are summarized in Table 3 in which both free binding energy (kcal/mol) and clash scores have been computed.

### 2.4. Inflammatory Mediators Assay

Inflammation is mediated by a variety of soluble factors [49,50,51,52], including a group of secreted polypeptides known as cytokines. Inflammatory cytokines [27] could be divided into two groups: those involved in acute inflammation (e.g., IL-1 (interleukin-1), TNF (tumor necrosis factor) and those responsible for chronic inflammation (cytokines mediating humoral responses IL-4, IL-5 and those mediating cellular responses IL-1, IL-2). Some cytokines, such as IL-1, significantly contribute to both acute and chronic inflammation. The role of IL-1 in inflammation [53,54] could be distinct to its own ability to trigger fever by enhancing prostaglandin E2 (PGE2) synthesis by the vascular endothelium of the hypothalamus and T cell proliferation stimulation. In addition, IL-1 elicited the release of histamine from mast cells at the site of inflammation. Histamine [50,55,56,57] then triggers early vasodilation and increase of vascular permeability. However, one of the first described [55,57,58,59] pharmacological roles of histamine has its ability to mimic anaphylaxis and has since been demonstrated to play a major role in inflammatory processes. In order to prevent inflammation, the mode of action of both inflammatory mediators and anti-inflammatory mediators must be well defined. Literature [60,61] has indicated that inflammatory cytokines affect the cells of the immune system migrating to the site of inflammation. Due to the action of inflammatory cytokines, cells tend to produce excessive amounts of inflammatory PGE2, COX-2, phospholipase, and others.

It is worth mentioning that inflammatory cytokines have stimulated [62,63,64,65] cells to synthesize other inflammatory cytokines, namely, IL-1β, TNF-α, IL-6, IL-8, and chemokines. The anti-inflammatory cytokines are a series of immune-regulatory molecules [34,50,66,67] that control the pro-inflammatory cytokine response. Anti-inflammatory cytokines have the ability to inhibit the synthesis of IL-1, tumor necrosis factor (TNF), and other major pro-inflammatory cytokines. Recently, in vivo and in vitro studies [68,69,70] showed that the effects of TNF-α have been parallel to or synergistic with those of IL-1β. However, the precise roles [69,70] that TNF-α and IL-1β play in pro-inflammation remain unknown.

## 3. Discussion

In the light of the above conclusions about the importance of pro-inflammatory cytokines and other inflammatory mediators in chronic disease, research is urgently needed to develop small molecules targeting cytokines to control their actions. Several NSAIDs have been known to affect the production or actions of cytokines [10,12,71,72,73] and this property has been considered to be a component of their actions, whether positive or negative. Yet, among NSAIDs, some might increase the production of interleukin-1 (IL-1β) or tumor necrosis factor (TNF-α) like indomethacin (nonselective inhibitor of COX-1 and COX-2) and these effects have been considered important in the development of GI (gastrointestinal) ulcers and asthma attributed to these drugs. However, other NSAIDs inhibit TNF-α, as nimesulide (a preferential COX-2 inhibitor), and ibuprofen (a nonselective COX-2 inhibitor) [74,75].

As it has been shown in this research, four compounds exhibiting the highest in vivo anti-inflammatory and best docking score (namely; **3c**, **3i**, **3j** and **3l**) have been tested for their effects on cytokines (namely; IL-1β, TNF-α), CRP, histamine and immunoglobulin E (IgE), comparing results with the standard drug diclofenac.

The results of inflammatory mediators confirmed those of in vivo anti-inflammatory and docking assays, as shown in Table 4. In general all compounds (**3c**, **3i**, **3j**, and **3l**), as well as diclofenac, significantly inhibit IgE compared with the control group. Compound **3l** showed the highest percentage of inhibition and top ranked docking score, exhibited a significant lower level of IL1-β and TNF-α compared with the control group. There is no significant difference in IgE and IL1-β levels between **3l** and diclofenac. Administration of compound **3i** showed a significantly lower level of histamine and TNF-α compared with the control group. IL-1β level of **3j**, was significantly lower than that in the control group. Compound **3c** was able to decrease CRP and TNF-α cytokine. All results and discussion are illustrated in details in Figure 4.

## 4. Materials and Methods

### 4.1. General Information

All commercial chemicals used as starting materials and reagents in this study have been purchased from Merck (Darmstadt, Germany) and were of reagent grade. All melting points are uncorrected and were measured using an Electrothermal IA 9100 apparatus (Shimadzu, Kyoto, Japan); IR spectra have been recorded as potassium bromide pellets on a Perkin-Elmer 1650 spectrophotometer (Waltham, MA, USA). ^1^H-NMR spectra were determined on a Mercury 300 MHz spectrometer (Varian, Cambridge, UK) and chemical shifts have been expressed as ppm against TMS as an internal reference. Mass spectra have been recorded at 70 eV on an EI MS-QP 1000 EX instrument (Shimadzu). Microanalyses have been performed using a Vario Elmentar apparatus (Shimadzu, Kyoto, Japan). Column chromatography has performed on silica gel 60 (particle size 0.06–0.20 mm, Merck). Compounds **1** and **2** have prepared as reported in literature [26,27,28,30]. The structures of all new compounds prepared in this paper have been confirmed by their spectral data.

### 4.2. Synthesis

#### 4.2.1. General Procedure for the Synthesis of Compounds **3a**–**l**

To a solution of **1a**–**f** (0.1 mol) in ethanol (30 mL), an appropriate arylidenemalononitrile (benzylidine or *p*-methoxybenzylidenemalononitrile, 0.1 mol) and piperidine (2 mL) were added. The reaction mixture was refluxed for 8 h. The mixture was left to cool at room temperature then poured onto crushed ice, and neutralized with hydrochloric acid. The solid precipitate was filtered off, washed with water, and crystallized from ethanol to yield **3a**–**l**.

*4-Amino-5-cyano-3,6-diphenyl-1-(4-methylphenyl)-1H-pyrrolo[2,3-b]pyridine* (**3a**). Yield: 80%; m.p.: 203–205 °C; IR (KBr) υ (cm^−1^): 3467, 3426 (NH_2_), 2176 (CN), 1576 (C=N); MS (EI) *m*/*z*: 400 (M+, 52%); ^1^H-NMR (DMSO-*d*_6_) δ (ppm): 2.4 (s, 3H, CH_3_), 4.9 (s, 2H, NH_2_, D_2_O exchangeable), 6.8–7.9 (m, 15H, Ar-H); Anal. Calcd. for C_27_H_20_N_4_ (400.47): C, 81.00; H, 5.00; N, 14.00%. Found: C, 80.86; H, 5.18; N, 14.26%.

*4-Amino-5-cyano-3,6-diphenyl-1-(4-methoxyphenyl)-1H-pyrrolo[2,3-b]pyridine* (**3b**). Yield: 94%; m.p.: 189–191 °C; IR (KBr) υ (cm^−1^): 3426, 3411 (NH_2_), 2215 (CN), 1597 (C=N); MS (EI) *m*/*z*: 416 (M+, 43%); ^1^H-NMR (DMSO-*d*_6_) δ (ppm): 3.54 (s, 3H, OCH_3_), 5.1 (s, 2H, NH_2_, D_2_O exchangeable), 6.9–7.9 (m, 15H, Ar-H); Anal. Calcd. for C_27_H_20_N_4_O (416.47): C, 77.88; H, 4.81; N, 13.46%. Found: C, 77.81; H, 4.59; N, 13.21.

*4-Amino-5-cyano-3,6-diphenyl-1-(1,5-dimethyl-3-oxo-2-phenyl-2,3-dihydro-1H-pyrazol -4-yl)-1H-pyrrolo[2,3-b]pyridine* (**3c**). Yield: 78%; m.p.: 182–184 °C; IR (KBr) υ (cm^−1^): 3445, 3417 (NH_2_), 2203 (CN), 1713 (C=O), 1588 (C=N); MS (EI) *m*/*z*: 496 (M+, 12.6%); ^1^H-NMR (DMSO-*d*_6_) δ (ppm): 2.21 (s, 3H, CH_3_), 3.4 (s, 3H, NCH_3_), 5.0 (s, 2H, NH_2_, D_2_O exchangeable), 7.1–8.0 (m, 16H, Ar-H); Anal. Calcd. for C_31_H_24_N_6_O (496.56): C, 75.00; H, 4.84; N, 16.94%. Found: C, 75.18; H, 4.66; N, 16.72%.

*4-Amino-5-cyano-2,3,6-triphenyl-1-(3,4-dichlorophenyl)-1H-pyrrolo[2,3-b]pyridine* (**3d**). Yield: 57%; m.p.: 134–136 °C; IR (KBr) υ (cm^−1^): 3425, 3418 (NH_2_), 2211 (CN), 1588 (C=N); MS (EI) *m*/*z*: 530 (M+, 26%; M + 2, 11%; M + 4, 2.2%); ^1^H-NMR (DMSO-*d*_6_) δ (ppm):5.21 (s, 2H, NH_2_, D_2_O exchangeable), 7.0–8.1 (m, 18H, Ar-H); Anal. Calcd. for C_32_H_20_Cl_2_N_4_ (531.43): C, 72.45; H, 3.77; Cl, 13.21; N, 10.57%. Found: C, 72.19; H, 3.46; Cl, 13.46; N, 10.71%.

*4-Amino-5-cyano-2,3,6-triphenyl-1-(4-methoxyphenyl)-1H-pyrrolo[2,3-b] pyridine* (**3e**). Yield: 88%; m.p.: 234–236 °C; IR (KBr) υ (cm^−1^): 3365, 3322 (NH_2_), 2219 (CN), 1618 (C=N), 1245 (C-O); MS (EI) *m*/*z*: 492 (M+, 54%); ^1^H-NMR (DMSO-*d*_6_) δ (ppm): 3.6 (s, 3H, OCH_3_), 4.9 (s, 2H, NH_2_, D_2_O exchangeable), 6.8–8.0 (m, 19H, Ar-H); Anal. Calcd. for C_33_H_24_N_4_O (492.57): C, 80.49; H, 4.88; N, 11.38%. Found: C, 80.15; H, 5.04; N, 11.71%.

*4-Amino-5-cyano-2,3,6-triphenyl-1-(1,5-dimethyl-3-oxo-2-phenyl-2,3-dihydro-1H-pyrazol-4-yl)-1H-pyrrolo[2,3-b]pyridine* (**3f**). Yield: 39%; m.p.: 172–174 °C; IR (KBr) υ (cm^−1^): 3332, 3316 (NH_2_), 2220 (CN), 1698 (C=O), 1605 (C=N); MS (EI) *m*/*z*: 572 (M+, 30.4%); ^1^H-NMR (DMSO-*d*_6_) δ (ppm): 2.25 (s, 3H, CH_3_), 3.5 (s, 3H, NCH_3_), 5.21 (s, 2H, NH_2_, D_2_O exchangeable), 6.8–8.0 (m, 20H, Ar-H); Anal. Calcd. for C_37_H_28_N_6_O (572.66): C, 77.62; H, 4.90; N, 14.69%. Found: C, 77.91; H, 4.96; N, 14.45%.

*4-Amino-5-cyano-3-phenyl-1-(4-methylphenyl)-6-(4-methoxyphenyl)-1H-pyrrolo [2,3-b]pyridine* (**3g**). Yield: 71%; m.p.: 208–210 °C; IR (KBr) υ (cm^−1^): 3434, 3397 (NH_2_), 2210 (CN), 1608 (C=N), 1219 (C-O); MS (EI) *m*/*z*: 430 (M+, 53%); ^1^H-NMR (DMSO-*d*_6_) δ (ppm): 2.3 (s, 3H, CH_3_), 3.5 (s, 3H, OCH_3_), 5.1 (s, 2H, NH_2_, D_2_O exchangeable), 6.9–7.9 (m, 14H, Ar-H); Anal. Calcd. for C_28_H_22_N_4_O (430.50): C, 78.14; H, 5.12; N, 13.02%. Found: C, 77.95; H, 5.36; N, 12.84%.

*4-Amino-5-cyano-3-phenyl-1,6-di(4-methoxyphenyl)-1H-pyrrolo[2,3-b]pyridine* (**3h**). Yield: 68%; m.p.: 202–204 °C; IR (KBr) υ (cm^−1^): 3419, 3382 (NH_2_), 2217 (CN), 1616 (C=N), 1234 (C-O); MS (EI) *m*/*z*: 446 (M+, 18.7%); ^1^H-NMR (DMSO-*d*_6_) δ (ppm): 3.51 (s, 3H, OCH_3_), 3.7 (s, 3H, OCH_3_), 5.2 (s, 2H, NH_2_, D_2_O exchangeable), 6.9–8.1 (m, 14H, Ar-H); Anal. Calcd. for C_28_H_22_N_4_O_2_ (446.50): C, 75.34; H, 4.93; N, 12.56%. Found: C, 75.65; H, 5.16; N, 12.64%.

*4-Amino-5-cyano-3-phenyl-6-(4-methoxyphenyl)-1-(1,5-dimethyl-3-oxo-2-phenyl-2,3-dihydro-1H-pyrazol-4-yl)-1H-pyrrolo[2,3-b]pyridine* (**3i**). Yield: 63%; m.p.: 195–193 °C; IR (KBr) υ (cm^−1^): 3387, 3356 (NH_2_), 2208 (CN), 1716 (C=O), 1605 (C=N), 1227 (C-O); MS (EI) *m*/*z*: 526 (M+, 37.5%); ^1^H-NMR (DMSO-*d*_6_) δ (ppm): 2.32 (s, 3H, CH_3_), 3.5 (s, 3H, OCH_3_), 3.7 (s, 3H, NCH_3_), 4.81 (s, 2H, NH_2_, D_2_O exchangeable), 6.9–8.2 (m, 15H, Ar-H); Anal. Calcd for C_32_H_26_N_6_O_2_ (526.59): C, 73.00; H, 4.94; N, 15.97%. Found: C, 72.76; H, 5.23; N, 15.71%.

*4-Amino-5-cyano-2,3-diphenyl-1-(3,4-dichlorophenyl)-6-(4-methoxyphenyl)-1H-pyrrolo[2,3-b]pyridine* (**3j**). Yield: 41%; m.p.: 154–156 °C; IR (KBr) υ (cm^−1^): 3314, 3293 (NH_2_), 2205 (CN), 1568 (C=N), 1234 (C-O); MS (EI) *m*/*z*: 560 (M+, 100%; M + 2, 65%; M + 4, 12.8%); ^1^H-NMR (DMSO-*d*_6_) δ (ppm): 3.57 (s, 3H, OCH_3_), 4.7 (s, 2H, NH_2_, D_2_O exchangeable), 6.8–7.9 (m, 17H, Ar-H); Anal. Calcd. for C_33_H_22_Cl_2_N_4_O (561.46): C, 70.71; H, 3.93; N, 10.00%. Found: C, 70.93; H, 4.13; N, 10.24%.

*4-Amino-5-cyano-2,3-diphenyl-1,6-di(4-methoxyphenyl)-1H-pyrrolo[2,3-b]pyridine* (**3k**). Yield: 82%; m.p.: 231–233 °C; IR (KBr) υ (cm^−1^): 3274, 3245 (NH_2_), 2224 (CN), 1603 (C=N), 1235 (C-O); MS (EI) *m*/*z*: 522 (M+, 36%); ^1^H-NMR (DMSO-*d*_6_) δ (ppm): 3.62 (s, 3H, OCH_3_), 3.7 (s, 3H, NCH_3_), 5.0 (s, 2H, NH_2_, D_2_O exchangeable), 6.9–8.0 (m, 18H, Ar-H); Anal. Calcd. for C_34_H_26_N_4_O_2_ (522.60): C, 78.16; H, 4.98; N, 10.73%. Found: C, 78.45; H, 5.33; N, 10.89%.

*4-Amino-5-cyano-2,3-diphenyl-6-(4-methoxyphenyl)-1-(1,5-dimethyl-3-oxo-2-phenyl-2,3-dihydro-1H-pyrazol-4-yl)-1H-pyrrolo[2,3-b]pyridine* (**3l**). Yield: 87%; m.p.: 186–188 °C; IR (KBr) υ (cm^−1^): 3367, 3321 (NH_2_), 2211 (CN), 1723 (C=O), 1598 (C=N), 1233 (C-O); MS (EI) *m*/*z*: 602 (M+, 67%); ^1^H-NMR (DMSO-*d*_6_) δ (ppm): 2.2 (s, 3H, CH_3_), 3.54 (s, 3H, OCH_3_), 3.6 (s, 3H, NCH_3_), 4.9 (s, 2H, NH_2_, D_2_O exchangeable), 6.9–8.0 (m, 19H, Ar-H); Anal. Calcd. for C_38_H_30_N_6_O_2_ (602.68): C, 75.75; H, 4.98; N, 13.95%. Found: C, 75.79; H, 5.12; N, 13.61%.

#### 4.2.2. General Procedure for the Synthesis of Compounds **4a**–**l**

The appropriate aminopyrrolopyridine **3a**–**l** (0.01 mol) was heated in formic acid (20 mL, 85%) under reflux for 3 h, cooled, poured onto ice water to give a precipitate, filtered off, dried, and crystallized from ethanol to yield compounds **4a**–**l**.

*5,9-Diphenyl-7-(4-methylphenyl)-3H-pyrrolo[2,3-b]pyrido[4,3-d]pyrimidin-4-one* (**4a**). Yield: 82%; m.p.: 226–228 °C; IR (KBr) υ (cm^−1^): 3412 (NH), 1726 (C=O), 1596 (C=N); MS (EI) *m*/*z*: 428 (M+, 44%); ^1^H-NMR (DMSO-*d*_6_) δ (ppm): 2.34 (s, 3H, CH_3_), 6.8–7.9 (m, 15H, Ar-H), 8.1 (s, 1H, C-2 H), 8.3 (s, 1H, NH, D_2_O exchangeable); Anal. Calcd. for C_28_H_20_N_4_O (428.48): C, 78.50; H, 4.67; N, 13.08%. Found: C, 78.82; H, 4.41; N, 13.23%.

*5,9-Diphenyl-7-(4-methoxyphenyl)-3H-pyrrolo[2,3-b]pyrido[4,3-d]pyrimidin-4-one* (**4b**). Yield: 87%; m.p.: 214–216 °C; IR (KBr) υ (cm^−1^): 3510 (NH), 1719 (C=O), 1609 (C=N), 1234 (C-O); MS (EI) *m*/*z*: 444 (M+, 19.5%); ^1^H-NMR (DMSO-*d*_6_) δ (ppm): 3.5 (s, 3H, OCH_3_), 6.9–7.9 (m, 15H, Ar-H), 8.12 (s, 1H, C-2 H), 8.3 (s, 1H, NH, D_2_O exchangeable); Anal. Calcd. for C_28_H_20_N_4_O_2_ (444.48): C, 75.68; H, 4.50; N, 12.61%. Found: C, 75.87; H, 4.84; N, 12.33%.

*5,9-Diphenyl-7-(1,5-dimethyl-3-oxo-2-phenyl-2,3-dihydro-1H-pyrazol-4-yl)-3H-pyrrolo[2,3-b]pyrido[4,3-d]pyrimidin-4-one* (**4c**). Yield: 68%; m.p.: 219–221 °C; IR (KBr) υ (cm^−1^): 3451 (NH), 1706, 1724 (C=O), 1617 (C=N); MS (EI) *m*/*z*: 524 (M+, 35%); ^1^H-NMR (DMSO-*d*_6_) δ (ppm): 2.6 (s, 3H, CH_3_), 3.5 (s, 3H, NCH_3_), 6.9–7.8 (m, 16H, Ar-H), 8.2 (s, 1H, C-2 H), 8.5 (s, 1H, NH, D_2_O exchangeable); Anal. Calcd. for C_32_H_24_N_6_O_2_ (524.57): C, 73.28; H, 4.58; N, 16.03%. Found: C, 73.57; H, 4.24; N, 15.75%.

*5,8,9-Triphenyl-7-(3,4-dichlorophenyl)-3H-pyrrolo[2,3-b]pyrido[4,3-d]pyrimidin-4-one* (**4d**). Yield: 58%; m.p.: 177–179 °C; IR (KBr) υ (cm^−1^): 3379 (NH), 1707 (C=O), 1600 (C=N); MS (EI) *m*/*z*: 558 (M+, 43.5%; M + 2, 9.2%, M + 4, 3.1%); ^1^H-NMR (DMSO-*d*_6_) δ (ppm): 6.8–8.0 (m, 18H, Ar-H), 8.25 (s, 1H, C-2 H), 8.46 (s, 1H, NH, D_2_O exchangeable); Anal. Calcd. for C_33_H_20_Cl_2_N_4_O (559.44): C, 70.97; H, 3.58; Cl, 12.54; N, 10.04%. Found: C, 70.67; H, 3.76; Cl, 12.41; N, 9.79%.

*5,8,9-Triphenyl-7-(4-methoxyphenyl)-3H-pyrrolo[2,3-b]pyrido[4,3-d]pyrimidin-4-one* (**4e**). Yield: 76%; m.p.: 245–247 °C; IR (KBr) υ (cm^−1^): 3406 (NH), 1699 (C=O), 1586 (C=N), 1263 (C-O); MS (EI) *m*/*z*: 520 (M+, 72.3%); ^1^H-NMR (DMSO-*d*_6_) δ (ppm): 3.64 (s, 3H, OCH_3_), 6.9–8.0 (m, 19H, Ar-H), 8.19 (s, 1H, C-2 H), 8.3 (s, 1H, NH, D_2_O exchangeable); Anal. Calcd. for C_34_H_24_N_4_O_2_ (520.58): C, 78.46; H, 4.62; N, 10.77%. Found: C, 78.71; H, 4.35; N, 10.53%.

*5,8,9-Triphenyl-7-(1,5-dimethyl-3-oxo-2-phenyl-2,3-dihydro-1H-pyrazol-4-yl)-3H-pyrrolo[2,3-b]pyrido[4,3-d]pyrimidin-4-one* (**4f**). Yield: 61%; m.p.: 195–197 °C; IR (KBr) υ (cm^−1^): 3447 (NH), 1682, 1714 (C=O), 1603 (C=N); MS (EI) *m*/*z*: 600 (M+, 83.6%); ^1^H-NMR (DMSO-*d*_6_) δ (ppm): 2.71 (s, 3H, CH_3_), 3.7 (s, 3H, NCH_3_), 6.7–7.9 (m, 20H, Ar-H), 8.18 (s, 1H, C-2 H), 8.45 (s, 1H, NH, D_2_O exchangeable); Anal. Calcd. for C_38_H_28_N_6_O_2_ (600.67): C, 76.00; H, 4.67; N, 14.00%. Found: C, 75.72; H, 4.92; N, 13.85%.

*5-(4-Methoxyphenyl)-9-phenyl-7-(4-methylphenyl)-3H-pyrrolo[2,3-b]pyrido[4,3-d]pyrimidin-4-one* (**4g**). Yield: 73%; m.p.: 228–230 °C; IR (KBr) υ (cm^−1^): 3340 (NH), 1691 (C=O), 1617 (C=N), 1229 (C-O); MS (EI) *m*/*z*: 458 (M+, 16%); ^1^H-NMR (DMSO-*d*_6_) δ (ppm): 2.5 (s, 3H, CH_3_), 3.67 (s, 3H, OCH_3_), 6.8–7.9 (m, 14H, Ar-H), 8.1 (s, 1H, C-2 H), 8.34 (s, 1H, NH, D_2_O exchangeable); Anal. Calcd for C_29_H_22_N_4_O_2_ (458.51): C, 75.98; H, 4.80; N, 12.22%. Found: C, 75.76; H, 4.93; N, 11.85%.

*5,7-Di(4-methoxyphenyl)-9-phenyl-3H-pyrrolo[2,3-b]pyrido[4,3-d]pyrimidin-4-one* (**4h**). Yield: 78%; m.p.: 223–225 °C; IR (KBr) υ (cm^−1^): 3374 (NH), 1703 (C=O), 1602 (C=N), 1225 (C-O); MS (EI) *m*/*z*: 474 (M+, 43.6%); ^1^H NMR (DMSO-*d*_6_) δ (ppm): 3.45 (s, 3H, OCH_3_), 3.6 (s, 3H, OCH_3_), 6.8–7.8 (m, 14H, Ar-H), 8.13 (s, 1H, C-2 H), 8.4 (s, 1H, NH, D_2_O exchangeable); Anal. Calcd for C_29_H_22_N_4_O_3_ (474.51): C, 73.42; H, 4.64; N, 11.81%. Found: C, 73.63; H, 4.91; N, 11.59%.

*5-(4-Methoxyphenyl)-9-phenyl-7-(1,5-dimethyl-3-oxo-2-phenyl-2,3-dihydro-1H-pyrazol-4-yl)-3H-pyrrolo[2,3-b]pyrido[4,3-d]pyrimidin-4-one* (**4i**). Yield: 57%; m.p.: 227–229 °C; IR (KBr) υ (cm^−1^): 3336 (NH), 1706, 1724 (C=O), 1617 (C=N), 1238 (C-O); MS (EI) *m*/*z*: 554 (M+, 28.4%); ^1^H-NMR (DMSO-*d*_6_) δ (ppm): 2.5 (s, 3H, CH_3_), 3.56 (s, 3H, OCH_3_), 3.73 (s, 3H, NCH_3_), 6.8–8.1 (m, 15H, Ar-H), 8.2 (s, 1H, C-2 H), 8.5 (s, 1H, NH, D_2_O exchangeable); Anal. Calcd. for C_33_H_26_N_6_O_3_ (554.60): C, 71.48; H, 4.69; N, 15.16%. Found: C, 71.71; H, 4.80; N, 14.80%.

*5-(4-Methoxyphenyl)-8,9-diphenyl-7-(3,4-dichlorophenyl)-3H-pyrrolo[2,3-b]pyrido[4,3-d]pyrimidin-4-one* (**4j**). Yield: 52%; m.p.: 186–188 °C; IR (KBr) υ (cm^−1^): 3417 (NH), 1718 (C=O), 1596 (C=N), 1234 (C-O); MS (EI) *m*/*z*: 588 (M+, 73.2%; M+2, 29%; M+4, 6%); ^1^H-NMR (DMSO-*d*_6_) δ (ppm): 3.51 (s, 3H, OCH_3_), 7.0–7.8 (m, 17H, Ar-H), 8.23 (s, 1H, C-2 H), 8.43 (s, 1H, NH, D_2_O exchangeable); Anal. Calcd. for C_34_H_22_Cl_2_N_4_O_2_ (589.47): C, 69.39; H, 3.74; Cl, 11.90; N, 9.52%. Found: C, 69.51; H, 3.93; Cl, 11.62; N, 9.81%.

*5,7-Di(4-methoxyphenyl)-8,9-diphenyl-3H-pyrrolo[2,3-b]pyrido[4,3-d]pyrimidin-4-one* (**4k**). Yield: 66%; m.p.: 256–258 °C; IR (KBr) υ (cm^−1^): 3475 (NH), 1720 (C=O), 1592 (C=N), 1227 (C-O); MS (EI) *m*/*z*: 550 (M+, 56%); ^1^H NMR (DMSO-*d*_6_) δ (ppm): 3.5 (s, 3H, OCH_3_), 3.59 (s, 3H, OCH_3_), 7.0–7.9 (m, 18H, Ar-H), 8.2 (s, 1H, C-2 H), 8.41 (s, 1H, NH, D_2_O exchangeable); Anal. Calcd for C_35_H_26_N_4_O_3_ (550.60): C, 76.36; H, 4.73; N, 10.18. Found: C, 76.65; H, 4.93; N, 9.87%.

*5-(4-Methoxyphenyl)-8,9-diphenyl-7-(1,5-dimethyl-3-oxo-2-phenyl-2,3-dihydro-1H-pyrazol-4-yl)-3H-pyrrolo[2,3-b]pyrido[4,3-d]pyrimidin-4-one* (**4l**). Yield: 73%; m.p.: 204–206 °C; IR (KBr) υ (cm^−1^): 3388 (NH), 1713, 1729 (C=O), 1578 (C=N), 1218 (C-O); MS (EI) *m*/*z*: 630 (M+, 21.7%); ^1^H-NMR (DMSO-*d*_6_) δ (ppm): 2.4 (s, 3H, CH_3_), 3.62 (s, 3H, OCH_3_), 3.7 (s, 3H, NCH_3_), 6.9–8.0 (m, 19H, Ar-H), 8.14 (s, 1H, C-2 H), 8.33 (s, 1H, NH, D_2_O exchangeable); Anal. Calcd for C_39_H_30_N_6_O_3_ (630.69): C, 74.29; H, 4.76; N, 13.33%. Found: C, 73.88; H, 4.89; N, 13.54.

### 4.3. Anti-Inflammatory Activity

The anti-inflammatory effect of the newly synthesized compounds was evaluated in accordance with the carrageenan-induced paw edema method (Winter et al. [76,77]). Male albino Sprague-Dawley rats (150–175 g) have been used taking into accounts the international principles and local regulations concerning the care and the use of laboratory animals (Olfert et al. 1993) [78]. The animal had free access to a standard commercial diet and water, kept at rooms maintained at about 25 °C. Carrageenan-induced hind paw edema is the standard experimental model of acute inflammation. Carrageenan (Sigma-Aldrich Co., St. Louis, MO, USA) is the phlogistic agent of choice for testing anti-inflammatory drugs as it is not known to be antigenic and is devoid of apparent systemic effects.

Twelve groups of animals each consisting of five rats have been selected. The 1st group was treated with the vehicle and left as control while the 2nd one was given diclofenac sodium by oral route in a dose of 50 mg/kg body weight (reference standard) [79] and tested compounds (**3b**, **c**, **d**, **g**, **h**, **I**, **j**, **k**, **l** and **4k**) were orally administered at equimolar dose levels. After 30 min, acute inflammation was induced by sub-plantar injection of 0.1 mL of 1% suspension of carrageenan in the right hind paw of all rats. Hind foot-pad thickness was measured with a micrometer caliber before and at 1, 2, 3 and 4 h after carrageenan injection. Percent inhibition of the tested compounds and the standard drug was calculated in comparison with vehicle control (100%).

### 4.4. Docking and Modelling Methods

All compounds were built and saved as Mol2 files. The crystal structure of COX-2 enzyme complexed with indomethacin was downloaded from the Protein Data Bank (PDB: 4COX). The protein was loaded into Leadit 2.1.2 [80] and the receptor components were chosen by selection of chain A as a main chain which in complexed with indomethacin. Binding site was defined by choosing naproxen as a reference ligand to which all coordinates were computed. Amino acids within a 8.5 Å radius were selected in the binding site. All chemical ambiguities of residues were left as default. Ligand binding was driven by enthalpy (classic triangle matching). For scoring, all default settings were restored. Intra-ligand clashes were computed by using clash factor = 0.6. Maximum number of solutions per iteration = 200. Maximum of solution per fragmentation = 200. The base placement method was used as a docking strategy.

### 4.5. Determination of Inflammatory Mediators

#### 4.5.1. Determination of Immunoglobulin E (IgE)

The quantitative determination [81,82,83] of immunoglobulin E (IgE) concentration in serum was based on a solid phase enzyme-linked immunosorbant assay (ELISA) using a BioCheck kit, Cat. No. BC-1035 according to Ishizaka [81]. Serum, taken from the treated rats after carrageenan injection, was added to the IgE antibody coated microtiter wells and incubated at room temperature for 30 min, then wells were washed, and IgE antibody labeled HRP were added. After incubation at room temperature for 30 min. the wells were washed with water. A solution of TMB reagent was added and incubated for 20 min. at room temperature, resulting in the development of blue color. Then stop solution (HCl, 1 N) was added for stopping color development. The color change to yellow and measured spectrophotometrically at 450 nm using an ELISA reader (ELX808, BIO TEK, Winooski, Vermont, USA). The concentration of IgE for each sample was determined from the standard curve.

#### 4.5.2. Determination of Interleukin 1β (IL-1β)

The serum rat interleukin 1β (IL-1β) concentrations were determined [54,84] using an ELISA kit (WKEA Med Supplies, Changchun, China). Serum was added to IL-1β antibody coated microtiter wells and incubated for 30 min. at 37 °C. Wells were washed. HRP enzyme labeled IL-1β antibody was added to each well and incubated again for 30 min. then, washed. After that substrate was added and incubated for 15 min. at 37 °C. Reaction was stopped and the color change was measured spectrophotometrically at 450 nm using the BIO TEK ELX808 ELISA reader. The concentration of IL-1β in the samples was determined by comparing the O.D. of the samples to the standard curve.

#### 4.5.3. Determination of Tumor Necrosis Factor α (TNF-α)

Serum tumor necrosis factor α (TNF-α) [60,85] concentrations were determined using an ELISA kit (WKEA Med Supplies Cat. No. WH-110). Serum was added to TNF-α antibody coated microtiter wells and incubated for 30 min. at 37 °C. Wells were washed. HRP enzyme labeled TNF-α antibody was added to each well and incubated for 30 min. then, washed. The last steps were similar to those in the determination of interleukin 1β (IL-1β).

#### 4.5.4. Determination of C Reactive Protein (CRP)

C reactive protein was determined in serum based upon the reaction [58] between CRP and latex covalently bound antibodies against CRP using a Spectrum CRP TurbiLatex kit, Cat. No. 560001 according to Young) [86]. CRP values were determined photometrically at 540 nm using a STATLAB SZSL60-Spectrum instrument (Spectrum, Hannover, Germany)

#### 4.5.5. Determination of Histamine

Serum histamine levels were determined using an ELISA kit (WKEA Med Supplies, Cat. No. WH-844). Serum was added to histamine antibody coated microtiter wells and incubated for 30 min. at 37 °C. Wells were washed. HRP enzyme labeled histamine antibody was added to each well and incubated for 30 min. then, washed. The last steps were similar to those in the determination of interleukin 1β (IL-1β).

### 4.6. Statistical Analysis

Results have been expressed as the mean ± SE, and different groups have been compared using one way analysis of variance (ANOVA) followed by Tukey-Kramer test for multiple comparisons.

## 5. Conclusions

As a part of this initiative we have prepared some fused pyrrolopyridines **3a**–**k** and their pyrimidine derivatives **4a**–**k**, and investigated their activities as promising anti-inflammatory structures. The biological results revealed that both fused pyrrolopyridines (**3i** and **3l**) show promising activity, and are found to be promising anti-inflammatory agents, coinciding with their docking results. In vitro inflammatory mediators assay have also been examined and revealed good to moderate activities for compounds **3i** and **3l** along with compounds **3c** and **3j**. These findings provide guidance for the design and structural modifications of these derivatives for better anti-inflammatory activity, which is important for the development of a new class of anti-inflammatory drugs.

## Figures and Tables

**Table 1:** List of all synthesized compounds shown in Scheme 1.

Compound Nos.	Ar	X	R	Compound No.	Ar	X	R
**Ia**	3,4-dichlorophenyl	Ph	------	**3f**	antipyrinyl	Ph	H
**Ib**	*p*-methoxyphenyl	Ph	------	**3g**	*p*-tolyl	H	OCH_3_
**Ic**	antipyrinyl	Ph	------	**3h**	*p*-methoxyphenyl	H	OCH_3_
**IId**	*p*-tolyl	H	------	**3i**	antipyrinyl	H	OCH_3_
**IIe**	*p*-methoxyphenyl	H	------	**3j**	3,4-dichlorophenyl	Ph	OCH_3_
**IIf**	antipyrinyl	H	------	**3k**	*p*-methoxyphenyl	Ph	OCH_3_
**1a**	*p*-tolyl	H	------	**3l**	antipyrinyl	Ph	OCH_3_
**1b**	*p*-methoxyphenyl	H	------	**4a**	*p*-tolyl	H	H
**1c**	antipyrinyl	H	------	**4b**	*p*-methoxyphenyl	H	H
**1d**	*p*-tolyl	Ph	------	**4c**	antipyrinyl	H	H
**1e**	*p*-methoxyphenyl	Ph	------	**4d**	3,4-dichlorophenyl	Ph	H
**1f**	antipyrinyl	Ph	------	**4e**	*p*-methoxyphenyl	Ph	H
**2a**	------------	------	H	**4f**	antipyrinyl	Ph	H
**2b**	------------	------	OCH_3_	**4g**	*p*-tolyl	H	OCH_3_
**3a**	*p*-tolyl	H	H	**4h**	*p*-methoxyphenyl	H	OCH_3_
**3b**	*p*-methoxyphenyl	H	H	**4i**	antipyrinyl	H	OCH_3_
**3c**	antipyrinyl	H	H	**4j**	3,4-dichlorophenyl	Ph	OCH_3_
**3d**	3,4-dichlorophenyl	Ph	H	**4k**	*p*-methoxyphenyl	Ph	OCH_3_
**3e**	*p*-methoxyphenyl	Ph	H	**4l**	antipyrinyl	Ph	OCH_3_

**Table 2:**
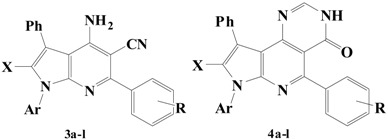
Anti-inflammatory activity of tested compounds, Results shown in paw size (mm, mean ± SD) and % inhibition over tested periods in comparison with control and standard drug.

Compound	1 h		2 h		3 h		4 h	
	Paw Size (mm)	%Inh	Paw Size (mm)	%Inh	Paw Size (mm)	%Inh	Paw Size (mm)	%Inh
**Control + ve**	5.86 ± 0.31	0	5.99 ± 0.43	0	6.11 ± 0.42	0	6.01 ± 0.46	0
**Diclofenac**	4.11 ± 0.19 ***	29.86	3.93 ± 0.21 ***	34.39	3.64 ± 0.20 ***	40.43	3.35 ± 0.27 ***	44.26
**3b**	5.09 ± 0.36 *	13.20	4.93 ± 0.51 **	17.71	4.69 ± 0.31 ***	23.24	4.61 ± 0.27 ***	23.30
**3c**	5.49 ± 0.37	6.31	4.81 ± 0.32 ***	19.70	4.76 ± 0.12 ***	22.10	4.61 ± 0.34 ***	23.29
**3d**	5.36 ± 0.34	8.53	5.62 ± 0.46	6.18	5.76 ± 0.38	5.73	5.63 ± 0.48	6.32
**3g**	5.49 ± 0.17	6.31	5.41 ± 0.24	9.68	5.40 ± 0.47	11.62	5.32 ± 0.48	11.48
**3h**	4.91 ± 0.25 **	16.21	4.73 ± 0.43 ***	21.04	4.71 ± 0.46 ***	22.91	4.63 ± 0.48 ***	22.96
**3i**	4.59 ± 0.56 ***	21.67	4.43 ± 0.42 ***	26.04	4.32 ± 0.39 ***	29.30	4.13 ± 0.53 ***	31.28
**3j**	5.69 ± 0.41	2.90	4.86 ± 0.40 **	18.87	4.55 ± 0.52 ***	25.53	4.21 ± 0.43 ***	29.95
**3k**	5.44 ± 0.25	7.17	5.39 ± 0.37	10.02	5.34 ± 0.52	12.60	5.18 ± 0.50	13.81
**3l**	4.69 ± 0.28 ***	19.97	4.19 ± 0.24 ***	30.05	3.89 ± 0.37 ***	36.33	3.81 ± 0.34 ***	36.61
**4k**	5.51 ± 0.34	5.97	5.47 ± 0.32	8.69	5.36 ± 0.31	12.28	5.14 ± 0.43	14.48

Paw size (mm) (Mean ± SE) *** *p* ˂ 0.001 (Highly Significant), ** *p* ˂ 0.01 (Moderate significant), * *p* ˂ 0.05 (Significant); $\%\;\mathrm{inhibition}=\frac{\left[\mathrm{Control}-\mathrm{Tested}\right]}{\left[\mathrm{Control}\right]}\times 100$.

**Table 3:** Free binding energy and clash score of all tested compounds and Diclofenac (standard COX Inhibitor).

Compound	ΔG Free Binding Energy (Kcal/mol)	Clash	Interacted Residues
**Diclofenac**	−16.85	5.2	*Tyr 355*
**3b**	−10.70	3.91	*Try 355* and *Ser 530*
**3c**	−10.12	3.11	*Ser 530* and *Tyr 355*
**3d**	−7.95	7.47	*Tyr 355*
**3g**	−7.06	7.21	*Arg 120*
**3h**	−10.52	3.45	*Arg 120*
**3i**	−11.25	3.50	*Tyr 385* and *Ser 530*
**3j**	−11.42	2.87	*Tyr 355*
**3k**	−7.62	6.95	*Ser 119*
**3l**	−12.23	2.51	*Ser 530* and *Tyr 355*
**4k**	−8.01	7.72	*Ser 119* and *Tyr 115*

**Table 4:** Active compounds against inflammatory mediators in comparison with diclofenac.

Active Compound/Drug	Histamine (μg/L)	IgE (IU/mL)	CRP (mg/L)	IL1-β (ng/L)	TNF-α (pg/mL)
**Control**	0.936 ± 0.024	3.7 ± 0.07	10.4 ± 0.89	11.8 ± 0.06	28.83 ± 0.19
**Diclofenac**	0.732 ± 0.009	1.7 ± 0.31 ***	7.6 ± 0.51 *	8.9 ± 0.05 *	26.28 ± 1.46
**3c**	0.784 ± 0.016	1.5 ± 0.28 ***	6.6 ± 0.80 *	11.58 ± 0.42	31.1 ± 0.10
**3i**	0.338 ± 0.195 **	1.68 ± 0.06 ***	10.8 ± 0.0.37	11.18 ± 0.90	20.3 ± 1.42 *
**3j**	0.762 ± 0.114	2.14 ± 0.05 ***	11.4 ± 1.20	7.44 ± 0.89 ***	26.88 ± 3.25
**3l**	0.819 ± 0.05	1.5 ± 0.07 ***	12.4 ± 0.51	7.6 ± 0.34 ***	16.1 ± 1.50 **

*** *p* ˂ 0.001 (Highly Significant), ** *p* ˂ 0.01 (Moderate significant), * *p* ˂ 0.05 (Significant); Result = (Mean ± SE).
